# Miscellaneous

**Published:** 1889-08

**Authors:** 


### MISCELLANEOUS.

Medicinal Value of Vegetables.—The qualities of different vegetables are worth knowing in the spring. According to one authority, celery acts upon the nervous system, and it is a cure for rheumatism and neuralgia. Tomatoes stimulate the liver; and spinach and common dandelion, prepared in the same way, have a direct effect on diseases of the kidneys. Onions, garlic and olives promote digestion by stimulating the circulatory system, with the consequent increase of the saliva and gastric juice. Raw onions are also regarded as a remedy for sleeplessness, and the French believe that onion soup is an excellent tonic in cases of debility of the digestive organs.

How to Clean Carpets.—For a carpet of about twenty yards or so take a pound of hard, white castile soap and scrape fine, add a quarter of a pound of washing soda, and as much spirits of turpentine as will bring it to the consistency of dough; make it into a ball. When it is time to clean the carpet take a pail of clear hot water and a large flannel cloth; wet the carpet with the flannel, then rub over with the ball of soap, and wipe off the soap with the flannel wrung as dry as possible. If the carpet is very much soiled a scrub brush may be used after the soap is applied. Clean about three-quarters of a yard at a time, and let it become thoroughly dry before it is used. Brussels and velvet carpets may be washed while on the floor, but great care should be taken not to let the water soak through.—*Detroit Free Press.*

Buttermilk is again coming to the front as food and medicine. Buttermilk and skim milk, half and half, have been used successfully in the treatment of dropsy, of heart disease, of Bright's disease, and of dyspepsia. No other food, moderate exercise, and go early to bed. In some of the cases so treated successfully, the patients were old chronic cases. One gentleman remarked that his endurance and ability to labor at his desk was really increased soon after the beginning of the treatment.—*The Clinical Reporter.*

Dennin's Certain Cure.—We would advise such of our readers as are afflicted with rheumatism or gout, to give the above remedy a fair trial. Having been advised of a number of cures through this means, we do not hesitate to recommend it. [See advertisement on another page.—Ed.]

The New York Pharmacal Association.—Is one of the most reliable concerns engaged in the preparation of medicines for the suffering. Its Lactopeptine is regarded, even by the profession, as a most valuable agent in overcoming weakness and restoring the human organism to a healthy condition.

One Of The Rights Of Women.—Bonaparte once at a party placed himself directly before a witty and beautiful lady, and said very abruptly, "Madam, I don't like that women should meddle with politics." "You are very right, General," she replied; 'but in a country where women are beheaded, it is natural that they should desire to know the reason."

Flowers.—Flowers are not trifles, as one might know, if he would only think how much pains God has taken with them everywhere—not one unfinished, not one bearing the marks of brush or pencil. Fringing the eternal borders of mountain winters—gracing the pulseless breast of the old gray granite; everywhere they are humanizing. Murderers do not ordinarily wear roses in their button-holes. Villians seldom train vines over cottage doors.—B. F. TAYLOR.

THE TRUE WIFE.—Oftentimes I have seen a tall ship glide by against the tide as if drawn by some invisible bowline, with a hundred strong arms pulling it. Her sails were unfilled, her streamers were drooping, she had neither side-wheel nor stern-wheel; still she moved on stately, in serene triumph, as with her own life. But I knew that on the other side of the ship, hidden beneath the great bulk that swam so majestically, there was a little toilsome steam-tug, with a heart of fire and arms of iron, that was tugging it bravely on; and I knew if the little steam-tug untwined her arm, and left the ship, it would wallow and roll about, and drift hither and thither, and go off with the refluent tide, no man knows whither. And so I have known more than one genius high-decked, full freighted, full sailed, gay pennoned, but that for the bare, toiling arms, and brave, warm-beating heart of the faithful little wife that nestles close to him, so that no wind or wave could part them, would have gone down the stream, and have been heard of no more.—O. W. HOLMES.

STERILIZED MILK.—Experiments with milk show that after it has been boiled and properly bottled, or otherwise protected from the germs which are always present in the air, it may be kept at the ordinary temperature for an indefinite time without spoiling. Milk which had thus been kept for three weeks could not be detected from milk which was perfectly fresh. Germany seems to have adopted the use of sterilized milk. There are large establishments in that country wholly devoted to the business of sterilizing milk. The bottles are first disinfected by the use of steam, and then the milk is strained into them through clean linen as soon as it is milked, the hands of the milkmen and the udders of the cows having been carefully cleansed with antiseptics. A distinguished physician, speaking of the benefits to be derived from the use of sterilized milk, states that he has employed it as a means of preventing summer diarrhea, and has never known of a single instance in which the disease has followed the use of milk freed from germs by the process described.

INGROWING NAILS.—Various remedies have been proposed, but probably nothing equals the application of carbolic acid. Brush the acid lightly over the inflamed surface and allow it to penetrate under the skin. In 24 hours it will be found that the nail is partly softened, and can be removed without pain, while the acid has acted as a complete anæsthetic to the tender inflamed tissue. Applied in the same way to a sprain or a bruise, in which the skin is unbroken, it affords instant relief.—*Daniel's Medical Jour.*

### THE BEST PRAYER.

He prayeth best who loveth best
 All things, both great and small;
For the dear God who loveth us,
 He made and loveth all. COLERIDGE.

